# Decoding populations in the ocean microbiome

**DOI:** 10.1186/s40168-024-01778-0

**Published:** 2024-04-01

**Authors:** Ramiro Logares

**Affiliations:** Institute of Marine Sciences (ICM), CSIC, Barcelona, Catalonia 08003 Spain

**Keywords:** Microbes, Populations, Ocean, Metagenomics, Metatranscriptomics

## Abstract

Understanding the characteristics and structure of populations is fundamental to comprehending ecosystem processes and evolutionary adaptations. While the study of animal and plant populations has spanned a few centuries, microbial populations have been under scientific scrutiny for a considerably shorter period. In the ocean, analyzing the genetic composition of microbial populations and their adaptations to multiple niches can yield important insights into ecosystem function and the microbiome's response to global change. However, microbial populations have remained elusive to the scientific community due to the challenges associated with isolating microorganisms in the laboratory. Today, advancements in large-scale metagenomics and metatranscriptomics facilitate the investigation of populations from many uncultured microbial species directly from their habitats. The knowledge acquired thus far reveals substantial genetic diversity among various microbial species, showcasing distinct patterns of population differentiation and adaptations, and highlighting the significant role of selection in structuring populations. In the coming years, population genomics is expected to significantly increase our understanding of the architecture and functioning of the ocean microbiome, providing insights into its vulnerability or resilience in the face of ongoing global change.

Video Abstract

**Supplementary Information:**

The online version contains supplementary material available at 10.1186/s40168-024-01778-0.

## Ocean microbes are key for the functioning of the Earth's system

The ocean microbiome is one of the main engines of the biosphere [1]. A massive number of cells populates it, with global estimates indicating ~ 10^29^ prokaryotic cells and ~ 10^30^ viruses [2, 3]. In one milliliter of open ocean water, there are typically 10^3^ protists, 10^6^ prokaryotes, and 10^7^ viruses [4]. Microbes account for ~ 70% of the biomass in the ocean, representing ~ 4.2 gigatons of carbon [5]. This biomass may be distributed across approximately 10^10^ species [6] that belong to a wide array of phylogenetic lineages with a long diversification history [7]. The ocean microbiome is crucial in global biogeochemical cycles [1, 8]. In the sunlit surface, microbes are responsible for an important fraction of the total atmospheric carbon and nitrogen fixation [9–11], representing ~ 46% of the global primary productivity [12]. Ocean microbes also play a fundamental role in processing organic matter by recycling nutrients and carbon to support additional production and channeling organic carbon to upper trophic levels [11, 13, 14]. Prokaryotes (bacteria and archaea) and unicellular eukaryotes or protists (including marine fungi) are key components of the ocean microbiome and feature fundamental differences in cellular structure, feeding habits, metabolic diversity, growth rates, and behavior [15]. Prokaryotic metabolisms are diverse and have major roles in global biogeochemical cycles [1, 8]. In contrast, protists' metabolisms are less diverse, but instead, they show major innovations in morphology and behavior [15]. A substantial fraction of the ocean microbiome biomass seems to comprise protists (and fungi) [5], including many heterotrophic groups that transfer carbon from prokaryotes or other protists to upper trophic levels.

### What is the total diversity of the ocean microbiome?

This question has been addressed in multiple works [6, 16–21] and so far, does not have a definitive answer. Current estimates of the total prokaryotic diversity on the planet vary significantly, with some differing by orders of magnitude [6, 19–21]. Nevertheless, over the past 20 years, there has been significant progress in understanding and delimiting the diversity of the vast array of microorganisms in the ocean. This is partly a consequence of the omics revolution that allowed retrieving microbes directly from the environment. Pioneering surveys ~ 20 years ago pointed to a large diversity of microbial genes and taxa in the ocean [22]. Subsequent large-scale oceanographic campaigns, such as *Malaspina* [23], TARA Oceans [24], Bio-GO-SHIP [25], and GEOTRACES cruises [26], significantly expanded our comprehension of the magnitude of the ocean’s microbiome diversity. These campaigns indicated ~ 50,000–100,000 protists and ~ 10,000–35,000 bacterial “species” or taxonomic units [16, 27, 28] in the open ocean plankton using High Throughput DNA Sequencing (HTS). From the metabolic perspective, TARA Oceans, based on metagenomics, has cataloged ~ 47 million predominantly prokaryotic genes [29] and ~ 116 million eukaryotic genes [30] at the global-ocean plankton scale. Similarly, the *Malaspina* consortium reported ~ 4 million predominantly prokaryotic genes from the deep ocean plankton [31].

The previous estimates show substantial variability due to multiple factors, such as different species definitions, molecular markers or methodologies, cryptic species, microdiversity, and biased sampling. Several estimates are bound to the evolutionary divergence captured by the ribosomal RNA (rRNA) gene or functional genes, which may miss fine-grained diversity or could introduce biases. For example, the rRNA gene may not capture differences between microbial species or populations. Similarly, different microbial species may share identical regions of their genomes, and when focusing on those areas, species will be indistinguishable.

Microdiversity typically refers to the genetic variation within a microbial species (i.e., below the species level) [32]. This genomic variation can arise from the presence of accessory genes, duplicated genes, single nucleotide variants (SNVs), or structural variants (insertions or deletions larger than 50 bp) [33]. Such variation may be organized into different lineages that are adapted to specific environmental conditions. This range of intra-specific diversity is well-exemplified by *Prochlorococcus*, the most abundant photosynthetic organism in the ocean [34]. Within *Prochlorococcus*, clades adapted to distinct environmental conditions, such as the low-light and high-light clades, have been identified. Furthermore, *Prochlorococcus* encompasses a vast array of accessory genes, which can be differentially combined among genotypes, conferring multiple adaptations [34]. At a finer scale, SNVs point to an even more nuanced level of adaptation.

Traits, which are commonly defined as attributes or characteristics of an organism that directly impact its fitness [35], can be influenced by microdiversity. Different traits can be acquired through horizontal gene transfer (HGT) [36, 37] or lost due to gene deletions [38, 39]. Additionally, trait variation can arise from the optimization of pre-existing traits, such as through the accumulation of SNVs or gene duplication [40]. Larkin and Martiny [40] have compiled evidence of niche partitioning related to microdiversity across multiple traits in both free-living and host-associated microbes. They linked these findings to primary processes: trait acquisition (TA), mainly through HGT, and trait optimization (TO), for example, via SNV accumulation. Among the traits exhibiting niche partitioning based on microdiversity, some can be relevant for understanding the impacts of global change on the future distributions of marine microbes. These traits include tolerance to temperature (driven by TO), and the utilization of carbon substrates (TA) and nutrients (TA and TO) [40]. A changing ocean is expected to affect microbial niches [41–43]. Taking into account how traits may respond to evolving niches, that is, through trait optimization or trait acquisition, could enhance our understanding of how microbes will react to environmental change.

The fundamental niche is the full range of environmental conditions and resources an organism can use without considering biotic interactions [44]. Microdiversity contributes to the fundamental niche of a microbial species, which is expected to be larger than that of any single individual [40]. Given our limited knowledge of the microdiversity of most microbes, it is likely that their fundamental niches are underestimated. Microdiversity can also contribute to understanding and predicting the distributions, or realized niches, of marine microbes [40]. The realized niche is shaped by the environmental conditions, available resources, biotic interactions, dispersal, and historical contingencies [44]. For a specific microbial species, environmental changes may result in shifts in the relative abundance of different populations or variants. These shifts might not necessarily alter the species’ realized niche [40] and could be interpreted as one of the mechanisms of microbial species to cope with environmental variation. However, when the environmental variations surpass the limits of adaptability provided by the species' microdiversity, adaptive evolution could be initiated through TO or TA [40, 45, 46]. Therefore, comprehending the existing microdiversity within marine microbes is crucial not only for assessing a species’ potential adaptability to environmental changes but also for tracking the ongoing shifts in variant distribution driven by evolving niches.

Understanding microbial microdiversity in the ocean represents a critical challenge for the coming years. Increasing knowledge within this field will yield important insights into microbial spatiotemporal distributions [40, 47–53], ecological interactions [54, 55], ecosystem function and its maintenance in fluctuating environments [49, 56–58], and the species’ reactions to changing niches due to climate change [43, 45, 46, 49]. Currently, only a few studies investigating the impact of climate change on microbial distributions have taken into account microdiversity and adaptative mechanisms [43, 46, 49, 59]. This limits the accuracy of predictions about the changes in the fundamental and realized niches of marine microbes in the future ocean. In the case of ecologically crucial species such as *Prochlorococcus*, predictive models have yielded a range of results, some of which are in agreement and others that present contrasting forecasts [42]. Specifically, different models consistently suggest that *Prochlorococcus* will expand its distribution range in response to a warmer ocean, increasing its realized niche. However, models present conflicting results regarding the changes in abundance within warm, oligotrophic regions [42]. Incorporating microdiversity within models could be key to yielding more unified and accurate predictions. Recent advancements have been made in modeling the microdiversity of *Prochlorococcus*, notably through the development of a 'pangenome-scale metabolic model' [49]. Growth rates for ecotypes exhibiting different niches were predicted by this model, which corresponded with abundances in an Atlantic Ocean transect. This model could be implemented to predict the relative abundance, composition, and activity of ecotypes of *Prochlorococcus* and other species in diverse marine regions [49]. Building upon a similar framework, models could be developed to forecast microbial microdiversity in future marine environments.

### Microbial species and populations

Understanding the genetic variation within and between microbial species remains a formidable challenge. This knowledge is crucial for elucidating the links between microbial processes and ecosystem function, including species and population dynamics, ecological interactions, and adaptive traits [33, 40, 60–62]. To achieve this, it is essential to identify and traverse the boundaries between species and populations, considering the full spectrum of diversity within marine microbes. However, this endeavor brings us to the question **‘**What is a microbial species?’ While this topic has sparked considerable debate [63–66], it falls beyond the scope of this discussion. Nevertheless, multiple studies have shown that natural microbial communities are composed of genotypic clusters of closely related organisms [36, 63]. These clusters can show cohesive environmental associations and dynamics, distinguishing them from other coexisting clusters. Hence, microbial species could be broadly defined as cohesive genetic units composed of individuals ecologically more similar to themselves than to other units [36]. However, defining the specific levels of genomic similarity that distinguish different species is outside the scope of this work; extensive discussions on this topic are available elsewhere [63–66]. The previous species definition integrates genetics and ecology, providing a broad and flexible framework for discussing population genomics in this piece. Here, populations will be considered as groups of organisms from the same species that inhabit a particular location or ecological niche at a specific time [33]. A group of individuals from the same species characterized by high genetic similarity and distinguishable from other groups based solely on genetic differentiation will be referred to as a “genetic cluster”. Genetic clusters may correspond to populations, also known as “genetic populations” [67].

Speciation and diversification seem to require both divergent selection and gene flow barriers to occur [68]. Selective or adaptive diversification and speciation would align with the *Ecological Species Concept* (ESC), where natural selection drives the process of divergence toward different niches [69], which is the speciation mechanism Darwin envisioned. Adaptive diversification is anticipated to increase microdiversity reflecting niche adaptations and potentially giving rise to ecotypes. In this context, ecotypes are broadly defined as strains—operationally characterized as genetic variants within a species [70]—that are ecologically similar to one another [71]. In turn, the *Biological Species Concept* (BSC) [72] emphasizes the restrictions on gene flow as the main mechanism of diversification and speciation. It is not necessarily expected that strains or species that have diversified due to restrictions to gene flow will display differential adaptation. Recent research suggests that microbial speciation may be driven by divergent selection (ESC-related process) and species differentiation maintained by barriers to gene flow (BSC-related process) [68].

The interplay of selection and homologous recombination has been proposed as a mechanism to explain the spread of adaptive genes among sympatric genetic clusters or populations [36]. If homologous recombination is low and selection high for a given gene, then individuals with the selected gene are expected to increase in abundance due to clonal expansion taking over the entire population, leading to a *genome-wide selective sweep* (GWSS) [65]. This process purges genetic variation from populations [36, 65]. In turn, if recombination is high compared to selection, selectively advantageous genes are expected to be exchanged among different population members without purging diversity, leading to a *gene-specific selective sweep* (GSSS). In GWSSs, an adaptive gene is expected to appear in a specific genomic background (that is, a specific genotype comprising all genes, except the adaptive one under consideration). In turn, in GSSSs, the selected gene is anticipated to be present in different genomic backgrounds. While these scenarios are simplified, they offer valuable frameworks for understanding the genetic diversity and structure of marine microbial populations. Analysis of the dynamics of GWSSs and GSSSs could shed light on the effects of global change on marine microbes. For example, a trend of increasing GWSSs or GSSSs among microbial species over time and their effects on microdiversity could point to stronger selective pressures exerted by global change.

**Fig. 1:**
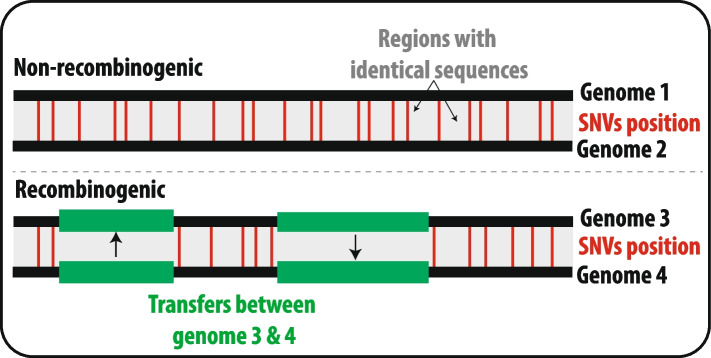
Microbial genomes that recombine (recombinogenic) and, therefore, belong to the same population or species would share longer identical regions than non-recombinogenic counterparts. Modified from Arevalo et al. [76]

When investigating microbial populations, one challenge is determining what organisms belong to the same species. One operative approach is to use genome similarity thresholds (e.g., the 95% threshold in the Average Nucleotide Identity [73, 74]) to delineate species. This is particularly useful in studies without multiple genomes from cultures to compare, as in marine metagenomics. Although these thresholds are practical and popular, they require an a priori decision on the cut-off level to delineate different operational taxonomic units (OTUs). The chosen threshold may or may not correspond with natural species. An alternative to using arbitrary thresholds is to search for natural discontinuities in genomic diversity that could reflect eco-genetic clusters representing populations or species. This approach has been referred to as *reverse ecology* [75, 76]. One example of its implementation is the methodology that uses recent gene flow to delineate eco-genetic units [75, 76]. Here, gene flow discontinuities are identified and used to delineate species (“gene flow units”) that can be subdivided into populations (“adaptively optimized gene flow clusters”) without using any prior environmental knowledge [76]. The rationale is that recent gene flow will leave a higher number of identical regions in genomes exchanging genes horizontally compared to what would be expected if mutations had accumulated without gene transfer [76] (Fig. 1). The reason is that horizontally exchanged DNA would not have had enough time to accumulate mutations compared to other regions shared by descent or vertically. Then, pairwise measurements of recent gene flow among genomes can be used to construct gene-flow networks to identify gene flow units (species) and gene flow clusters (populations) within them. This approach produced genome clusters corresponding to previously identified populations of *Vibrio*, *Sulfolobus,* and *Prochlorococcus* [76]. Results also indicated strong discontinuities in the gene flow between species (gene flow units), aligning with the Biological Species Concept [72]*.*

### From population genetics to population genomics

*Population genetics* investigate the ecological and evolutionary forces that generate, assort, and remove variation within species using specific marker genes or genomic areas. *Population genomics* represents an extension of population genetics that focuses on entire genomes [51]. While population genetics is a well-established field, population genomics is a relatively emerging field in environmental microbiology. Its growth has been promoted by decreasing DNA sequencing costs and the increasing popularization of long-read sequencing technologies. Population genomics has a huge potential for advancing our understanding of ocean microbes. It can bring new insights into their present and future spatiotemporal distributions, adaptations and contemporary evolution, metabolisms, ecological interactions, pathogenicity, as well as roles in ecosystem functioning and resilience to global change [33, 40, 45, 46, 49–51, 59, 60, 77].

A future challenge is to better comprehend the mechanisms underpinning the genetic makeup of marine microbial populations. The main forces determining the genetic composition of populations are *mutation*, *selection*, *gene flow*, and *genetic drift. Mutation* is the emergence of new and random gene variants and is the ultimate source of diversity. *Selection* changes allele frequencies due to their fitness impact on the phenotype, while *gene flow* is related to the exchange of genes between populations. Lastly, *genetic drift* refers to the random fluctuations in allele frequencies from one generation to the next due to the stochastic sampling of individuals contributing offspring to the next generation [78]. Even though microbial population genetics and genomics are growing fields [51, 79, 80], our understanding of *mutation*, *selection*, *gene flow*, and *genetic drift* is still predominantly based on the study of animals and plants. Comprehending how these processes operate in marine microbes is crucial, as microbes differ from multicellular organisms in at least three fundamental aspects: *dispersal*, *reproductive rates*, and *population size* [81, 82]. Even though the dispersal rate of most microbes, including those in marine environments, is still largely unknown, indirect evidence points to high dispersal rates [81, 83] that could be substantially higher than in multicellular organisms. While it has been argued that organisms with < 1 mm in size have virtually no barriers to dispersal [84], multiple studies during the last two decades point to dispersal limitation in microbes [28, 81, 83, 85]. Yet, detection limits in specific studies could have inflated the estimated dispersal limitation by not detecting low-abundance microbes that would be part of a large seed bank widely distributed in the global ocean [86, 87]. Furthermore, the reproductive rates of multicellular organisms tend to be lower than those of microbes. For example, generation times in small mammals can be in the order of months, while in some bacteria, it can be in the order of minutes/hours. Shorter generation times imply that mutation, adaptation, and divergence can occur faster in microbes than in multicellular organisms, which is particularly relevant for comprehending the possible contemporary adaptation of the ocean microbiome to climate change [45, 46]. Lastly, we must consider the census and effective population sizes to understand the genetic makeup and structure of marine microbial populations.

### Census vs. effective population size

Census (*N*) and effective population size (*N*_*e*_) are key parameters that can affect adaptation, drift, and dispersal. The census population size *N* refers to the total number of individuals or cells and can influence random dispersal as more individuals or cells increase the chances of arriving at new locations. In turn, the effective population size *N*_*e*_ represents the number of individuals in a theoretical population that would experience the same amount of genetic drift as the population under consideration. *N*_*e*_ plays a pivotal role in population genetics. It influences the magnitude of genetic drift, the extent of genetic variability within a population, and the balance between the efficacy of selection and the random effects of drift [78]. Specifically, a population's neutral genetic diversity, which refers to genetic variations without fitness effects, is estimated by the product of the effective population size *N*_*e*_ and the mutation rate. Furthermore, *N*_*e*_ is tied to the efficacy of selection. It dictates whether a beneficial mutation proliferates or a deleterious one is purged, with the outcome governed by the product of *N*_*e*_ and the intensity of selection [78]. Small *N*_*e*_ can increase genetic drift, which can lead to reduced genetic diversity over time, increase the likelihood of the fixation of deleterious alleles, and increase the chances of losing advantageous alleles [88].

While *N* can be huge in microbes, *N*_*e*_ is usually smaller due to the variance in reproductive success and potential selective sweeps. Lynch and colleagues calculated *N*_*e*_ ~ 10^5^ for vertebrates, ~ 10^6^ for invertebrates and land plants, ~ 10^7^ for unicellular eukaryotes, including fungi, and 10^8^ for free-living prokaryotes [89, 90]. These broad estimates suggest a higher genetic drift in large multicellular eukaryotes compared to prokaryotes, indicate that the effective population sizes are significantly smaller than the census population sizes, and imply that selection will be more efficient in large microbial populations than in multicellular counterparts [78]. Effective population sizes for prokaryotes show substantial variability, ranging between 10^6^ (host-associated) and 10^10^ (free-living), typically exceeding > 10^8^ [91, 92], while for microbial eukaryotes, *N*_*e*_ varies between 10^6^ (host-associated) and 10^8^ (free-living) [92].

*N*_*e*_ remains unknown for most marine microbial species [93], limiting our understanding of their adaptability to a changing ocean. Measuring the *N*_*e*_ of marine microbes could reveal unexpected results and change paradigms. For example, *Prochlorococcus* has an estimated average global abundance (*N*) of 3 × 10^27^ cells annually [41], yet its *N*_*e*_ has been recently calculated to be significantly smaller, approximately 1.7 × 10^7^ cells [94]. Furthermore, the estimated *N*_*e*_ was surprisingly smaller than that of other free-living bacteria, suggesting that drift could be a driver of evolution in this lineage [94]. Similarly, SAR11, which has a massive census population size of ~ 2.4 × 10^28^ [95], may have an effective population size smaller than that of *Roseobacter* [93]*.* Considering the crucial role of *N*_*e*_ in discerning the adaptive potential of marine microbial populations to climate change, it is imperative to determine this parameter, at least for those species with key roles in ocean ecosystem function.

### Microbial population diversity, structure, and adaptations in the omics era

Even though our understanding of the genomic diversity and structure of environmental microbial populations, as well as the genetic basis of ecotype differentiation, remains limited, the field is advancing rapidly. Studies focusing on the human microbiome [33] particularly demonstrate this advancement, though research on aquatic microbes is still less common. Nevertheless, previous works pointed to high genomic diversity and the presence of ecotypes within environmental microbial species. For example, ecotypes adapted to different light intensities [96], and temperatures [97] were found in the marine *Prochlorococcus*. Further studies indicated that *Prochlorococcus* includes an enormous population variation, with hundreds of strains coexisting in small seawater samples [34, 98]. These strains display a substantial allelic variation in their core genome (including housekeeping and ecologically relevant genes), delineating different genomic backbones. Each genomic backbone was linked to distinct sets of flexible genes that may reflect different metabolic functions, thus pointing to adaptive evolution [98]. In addition, a clear genomic differentiation was found between *Prochlorococcus* populations present in the Pacific and Atlantic oceans [53]. Populations of *Prochlorococcus* in the Pacific displayed greater diversity than those in the Atlantic, with no single population dominating. The populations from these two oceanic regions appear to comprise largely distinct groups with minimal overlap, each characterized by unique genomic backbones. Another study [36] compared the population divergence in marine strains of *Vibrio cyclitrophicus* [99] as well as in the hot-spring archaeon *Sulfolobus islandicus* [100]. Both species displayed divergent single nucleotide variants (SNVs) that tended to be concentrated in specific genomic areas. While in *Vibrio* the divergent SNVs tended to be localized in genomic “islands”, in *Sulfolobus* they tended to be spread across genomic “continents”. Outside these islands or continents, populations displayed a low divergence [36]. Genomic islands in *Vibrio* often contain ecologically relevant genes, suggesting that divergent SNVs are likely involved in ecological adaptation and were acquired by recombination.

**Fig. 2:**
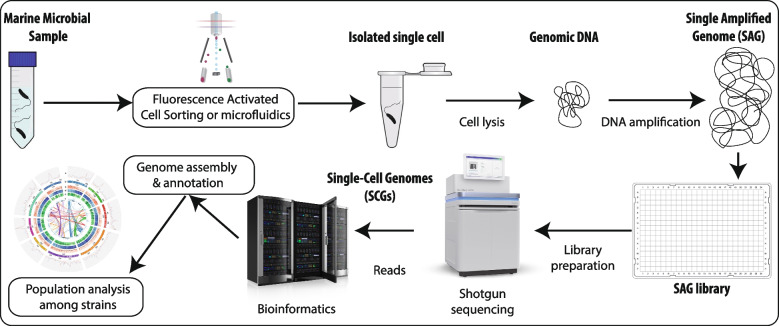
Single cell genomics [102]. In a nutshell, this approach starts with isolating single microbial cells, typically using fluorescence activated cell sorting (FACS) or microfluidics. Then, cells are lysed, and their genomic DNA is amplified, generating single amplified genomes (SAGs). SAGs are subsequently shotgun sequenced, and the produced reads (DNA sequences) are assembled and annotated. Those SAGs from the same species can then be used for population genomics analyses (as in Kashtan et al. [53, 98]). Furthermore, SAGs can be used as genomic templates in metagenome-based population genomics analyses [103] (Fig. 3)

**Fig. 3:**
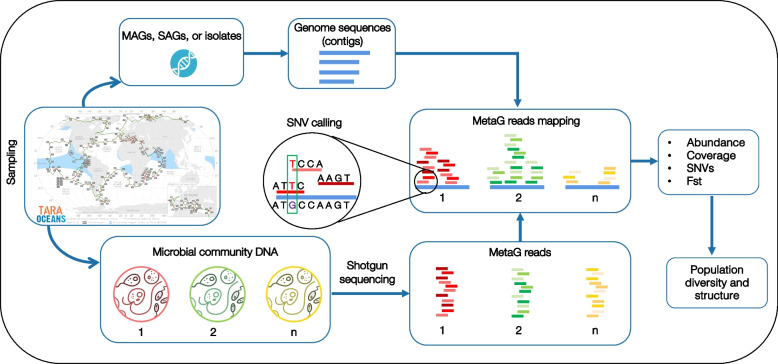
Metagenome-based population genomics [103]. Metagenome-assembled genomes (MAGs), Single Amplified Genomes (SAGs; Fig. 2), or genomes from isolates are generated after sampling or retrieved from collections. In parallel, marine metagenomes (MetaG) are produced from community DNA or retrieved from databases. Subsequently, unassembled metagenomes (reads) are mapped against MAGs, SAGs, or sequenced isolates. After mapping, the abundance and the horizontal and vertical coverages of each MAG, SAG, or isolate are calculated, and Single Nucleotide Variants (SNVs) are called. Based on the SNVs, population-level diversity, and structure (based on the Fst index) can be assessed. The trajectory of the TARA Oceans sampling campaign is shown as an example. See an application of this approach in Fig. 4

Several of the previous studies have used cultured microbial strains to investigate population diversity and structure. Yet, most of the microbial diversity cannot be cultured [101]. Therefore, culture-independent approaches have started to be used to investigate wild microbial populations, such as Single-Cell Genomics (Fig. 2) and Metagenomics [33, 98, 102, 103]. A number of studies have recently started to leverage the power of Metagenome-Based Population Genomics [33, 103] and the availability of large public metagenomic datasets to investigate microdiversity in aquatic microbes (Fig. 3). These metagenomic studies can be divided into two main classes: (1) those that compare metagenomic information against a collection of genomes or sequences of interest (e.g., POGENOM [104], MIDAS [105], metaSNV [106], StrainPhlAn [107], and inStrain [108]; Fig. 3) and (2) reference-free approaches that investigate fine-grained variation among metagenomic reads (e.g., metaVaR [109]). Metagenome-based population genomics studies frequently focus on the microdiversity associated with SNVs, therefore linked to the potential optimization of traits [40]. Furthermore, there are methods that aim at reconstructing strains or haplotypes from the metagenomic data (e.g., ConStrains [110], DESMAN [111], STRONG [112], InStrain [108], and Strain-GeMS [113]). Given the space limitations, below, I will provide a few examples of some of these approaches applied to marine microbes to convey the central message without aiming for a comprehensive review.

One pioneering study compared the information present in metagenomes against a compiled database of ca. 30,000 reference bacterial genomes using a tailored bioinformatics pipeline (MIDAS) [105]. This approach was used to investigate the population-level variation in 198 marine metagenomes from TARA Oceans coming from 66 stations in the global ocean [114]. Not surprisingly, it was found that, in general, the reference bacterial genomes used in MIDAS had low coverage in the ocean samples. Nevertheless, sufficient recruitment was evidenced for reference genomes of the genera *Pelagibacter*, *Alteromonas*, *Synechococcus*, and *Marinobacter* [105]*.* Pan-genome analyses showed a substantial variability of gene content in these species across the marine metagenomes. When all species were considered, an average of 19% of the genes differed between metagenomes [105], indicating significant variability in gene content between strains across marine stations. Based on the variability in gene content of each bacterial species, authors found that the populations of different species were grouped by ocean region. For instance, one SAR11 species (*Candidatus Pelagibacter* sp. genome HTCC7211) was segregated into three distinct clusters, each aligning with a specific geographic region: the Mediterranean Sea, the South Atlantic Ocean, and the South Pacific Ocean (note that these clusters point to populations of the specific SAR11 genome HTCC7211, and do not necessarily correspond to clusters identified in other SAR11 genomes). Each cluster encompassed samples from different water layers [105]. Furthermore, geographic distance decay in gene content was detected for most of the species examined. Hence, there appears to be a correlation between strain gene content and geographical distribution for several marine bacterial species.

As one of the most abundant lineages in the ocean, SAR11 [95] serves as an ideal model species for population genomics studies, facilitating the exploration of fine-grained microbial adaptations to the marine environment. SAR11 features sub-clades with specific ecological preferences and contains a large microdiversity [95, 115–117]. Large amounts of microdiversity and frequent recombination [118] seem to reduce the recovery of SAR11 contigs from metagenomes, even when the number of reads is high, which limits the number of recovered Metagenome-Assembled Genomes (MAGs) [52, 117]. The low recovery of MAGs complicates population genomics analyses, yet a number of studies have found ways to leverage the large amounts of SAR11 information in marine metagenomes. It is important to note that different studies, which utilize various isolates of SAR11, are likely examining population differentiation within distinct species originating from this highly diverse lineage. Haro-Moreno and colleagues investigated the diversity and distribution of SAR11 using a large collection of Single-Amplified Genomes (SAGs), cultures, and MAGs, together with a collection of 620 metagenomes [117]. A large population-level diversity was detected, indicating that this is a characteristic of Pelagibacterales. Furthermore, population-level diversity was conserved across a broad horizontal dimension of the ocean, pointing to a limited influence of horizontal biogeography in the structure of microdiversity for the investigated lineage. In turn, population-level diversity displayed marked changes across the water column at single locations. This indicates that the vertical dimension of the ocean has a larger impact on microdiversity than the horizontal, despite their large differences in geographic scale (a few kilometers vs. hundreds or thousands of kilometers, respectively). This study also reports many synonymous single nucleotide variants (SNVs) in the investigated genomes, which aligns with a strong purifying selection. Only a few genes displayed positive selection, which could be the basis of strain or population adaptation [117]. Similarly, Delmont and colleagues [52] examined the population variation of an abundant isolate of SAR11 in the surface global ocean using metagenomics and found a large amount of variation in terms of Single Amino-Acid Variants (SAAVs). More protein variants were detected in cold than in warm currents, suggesting different adaptive patterns in populations. Clustering metagenomes based on the SAAVs they feature (i.e., the potential populations that metagenomes represent) revealed two main SAR11 clusters corresponding to warm or cold large-scale ocean currents, suggesting two main niches for this SAR11 isolate [52]. At a finer scale, 6 proteotypes were identified, grouping samples with similar amino acid variants; these tended to display specific distributions in the global ocean linked to temperature, basins, and/or currents. Altogether, the correlation between SAR11 population-level diversity and environmental variables, particularly temperature, suggests that selection plays a more important role than dispersal in shaping the population structure of this key marine lineage. Another study provided evidence of two widespread populations within a SAR11 genome [106]: one population was predominantly found in the Atlantic, Indian, and North-Pacific oceans, while the other was mainly present in the South-Pacific Ocean. The correspondence between these populations and those reported in previous studies needs further investigation, given the different genomes that were analyzed.

Population-level variation correlating with environmental heterogeneity was also reported in a study of bacterioplankton in the Baltic Sea [104]. Here, Sjöqvist and colleagues investigated the population-level diversity and structure of 22 MAGs that were representative of genomic clusters by using metagenomes from a 1700-km transect and a time series. A substantial number of SNVs were detected for the 22 MAGs. Intra-sample mean nucleotide diversity (representing the probability that two metagenomic reads covering a genomic position differ) displayed specific patterns for some MAGs in the spatial dimension, while no temporal trends were observed [104]. Most MAGs displayed a non-random population structure across the Baltic Sea, as measured by the fixation index (Fst, a measure of population differentiation). Salinity and temperature emerged as the first and second spatial drivers of population structure, respectively [104]. In four MAGs, evidence of isolation by distance (geographic effects) was detected. Temporal temperature variation was a significant population structuring driver for two MAGs (out of the four that could be analyzed). Overall, population differentiation was higher across the Baltic Sea than temporally. This suggests that spatial differences in salinity and temperature are a stronger driver of population differentiation than seasonal variation of environmental variables. Differentially adapted genes were detected in populations present at different salinities, suggesting they may be the basis of population adaptation. In contrast to the global ocean, where temperature appears to be the central factor influencing population structure [52], this study [104] identifies salinity as the primary driver shaping populations in the Baltic Sea, a region characterized by substantial salinity gradients. Day length could also drive population structure in the Baltic Sea and should be tested in future studies, as this variable was shown to influence the structure of microbial communities [119, 120].

Metagenome-based population genomics approaches have also been used to investigate marine protists. Leconte and colleagues investigated the population genomics of the picophytoplankton *Bathycoccus* RCC1105 isolated in January 2006 from the SOLA station (Banyuls-sur-Mer, France) in the Western Mediterranean Sea at 3 m depth [121]. Broad population-level variation patterns were assessed using surface and deep chlorophyll maximum metagenomes from the TARA Oceans campaign corresponding to the 0.8–5-μm organismal size fraction. Of the original 162 TARA Oceans metagenomes, only 27 (ca. 17%) from diverse geographic locations and different ocean basins displayed enough coverage of the reference genome for downstream analyses [121]. Even though *Bathycoccus* has a relatively small genome (~ 15 Mb [122]) and displays widespread geographic distributions [123], the previous results evidence the greater difficulties of applying the metagenome-based population genomics approach to protists compared to prokaryotes [124]. The primary reason is that marine metagenomes generally encompass more prokaryotic than eukaryotic information, compounded by the inherently larger size and complexity of eukaryotic genomes. Nevertheless, when comparing the 27 metagenomes based on the SNVs they contain, it was found a clear separation between those originating from Arctic and temperate regions [121]. In addition, Arctic populations displayed a clear separation from Austral ones. A positive correlation between population and temperature differences was found [121], indicating, as in the previous example of SAR11, the relevant role of temperature in structuring the genomic variation of microbial populations in the ocean. Furthermore, 2742 SNVs and 13 SAAVs were detected that differentiate temperate from cold *Bathycoccus* populations. The structure of protein variants from mesophilic and psychrophilic populations was compared, which provided insights into the structural changes that may underpin adaptation to different temperature niches and that are responsible for changes in functional and physical properties [121].

In another work, Da Silva and colleagues investigated the genomic differentiation within three species of pico-phytoplankton in the Mediterranean Sea: *Bathycoccus prasinos*, *Pelagomonas calceolata*, and *Phaeocystis cordata* [124]. Here, metagenomic reads from TARA Oceans stations in the Mediterranean Sea were mapped to either reference genomes (*B. prasinos*), or transcriptomes (*P. calceolata* and *P. cordata*) retrieved from the Mediterranean Sea or other regions. In general, *B. prasinos* displayed a higher population differentiation than *P. calceolata* and *P. cordata* in the Mediterranean Sea. In addition, results indicated that environmental selection seems to shape the population-level diversity of *B. prasinos* in the Mediterranean Sea, while *P. cordata* populations appear to be shaped by geographic distance (isolation by distance) [124]. This study demonstrates that populations of different protist species within the same functional group and with similar morphologies can exhibit varying degrees of differentiation and be influenced by distinct mechanisms, such as selection versus dispersal.

The previously discussed metagenome-based population genomics studies required reference genomes or transcriptomes to map against metagenomic reads. This is a limitation, as there are no partial or complete genomes or transcriptomes for most microbial species at the moment. Therefore, alternative reference-free approaches have been developed, which do not need an alignment to a reference and can detect variants directly on unassembled metagenomic reads. One such approach is metaVaR, which introduces the concept of metavariant, which are variants detected in metagenomic reads [125]. Then, metavariant species, or MVS, can be defined by clustering metavariants. Thus, an MVS includes metavariants from the same species. MVSs can then be taxonomically assigned by aligning variable loci against sequence databases [125]. Despite the potential of this approach to investigate the population genomics of microbial species with no reference genomes, in reality, only a number of species are expected to present enough metagenomic coverage and the number of metavariants needed to pass the quality thresholds. For example, this approach was tested in a large dataset derived from TARA Oceans that included millions of metavariants from 114 geographically widespread marine samples, and only 113 MVSs were retrieved [125, 126]. The 113 MSVs belonging to Metazoa, Chromista, Chlorophyta, Bacteria, and viruses were analyzed across the North and South Atlantic Oceans, Southern Ocean, and the Mediterranean Sea [109]. Population differentiation (as measured by the Fst index) was higher among ocean basins than within basins for the analyzed species, which could be attributed to higher connectivity within basins. Furthermore, unicellular organisms (bacteria, unicellular eukaryotes, and viruses) displayed more population structure than larger multicellular counterparts (zooplankton). This could be attributable to different dispersal capabilities affecting gene flow or different demographic histories (population size, generation time). The primary drivers of population structure for the studied species were oceanic currents (Lagrangian travel time), temperature, and salinity [109]. Yet, in this work, a large fraction of the population genomic differentiation could not be explained, pointing to other abiotic (e.g., additional inorganic nutrients and pH) and biotic variables (ecological interactions) that could contribute to population structure [109]. All in all, this approach represents a valuable option for metagenome-based population genomics when no reference genomes are available. Still, this methodology does not intend to replace reference-based methods, which according to the authors, should be used whenever a reference is available [125].

Metatranscriptomics has been used to investigate multiple aspects of marine microbial communities, such as the variability in gene expression in the global surface ocean [29], diel and seasonal dynamics [127–130], metabolic functions in sinking particles [131], effects and degradation of pollutants [132, 133], detection of active functional groups [134], nutrient cycling [135], and expression of secondary metabolites [136]. Yet, few studies have applied metatranscriptomics to investigate gene expression among closely related strains or populations. By coupling predefined gene-OTUs and metatranscriptomics, Shilova and colleagues [137] found different gene transcription patterns in strains of *Synechococcus* and *Prochlorococcus*, *Pelagibacter,* and the photosynthetic picoeukaryote *Ostreococcus*. These findings support the hypothesis that strain-level genetic diversity within marine microbes can produce distinct gene expression patterns reflecting specific niches or adaptations. Another work found high intraspecific genomic diversity for intracellular bacterial symbionts of deep-sea mussels using a metagenome-based population genomics approach [138]. Then, metatranscriptomics showed that most of the strain-specific genes were expressed. This study demonstrates the utility of integrating metatranscriptomics with metagenome-based population genomics to determine the expression of intra-specific genetic variation that may manifest in phenotypic effects.

Epigenetics refers to heritable changes in gene expression that occur without alterations in the DNA sequence, primarily through mechanisms such as DNA methylation, histone modification, and non-coding RNA gene silencing [139]. Although epigenetic research has predominantly focused on animals and plants, the process is also widespread in microbes [139, 140], and its effects on their ecology and evolution are starting to be understood. Epigenetic modifications can lead to diverse microbial phenotypes and populations [139], affecting ecological and evolutionary processes like dormancy [141], parasitism [142], and adaptive diversification [140]. Recent research in marine bacteria has revealed a significant positive correlation between variations in the methylome and population-level genomic differentiation [143]. This suggests that genetic and epigenetic variations may synergistically influence the divergence of populations. Another study identified distinct thermal ecotypes in genetically identical marine *Synechococcus*, attributed to variations in methylation sites [60]. While the analyzed *Synechococcus* ecotypes shared 436 methylation sites, most were strain-specific. Nevertheless, the methylated genes were found to be part of similar KEGG modules [60]. These works emphasize the role of epigenetics in the study of microbial populations, potentially revealing locally adapted strains or ecotypes with identical or nearly identical genomic composition.

**Fig. 4:**
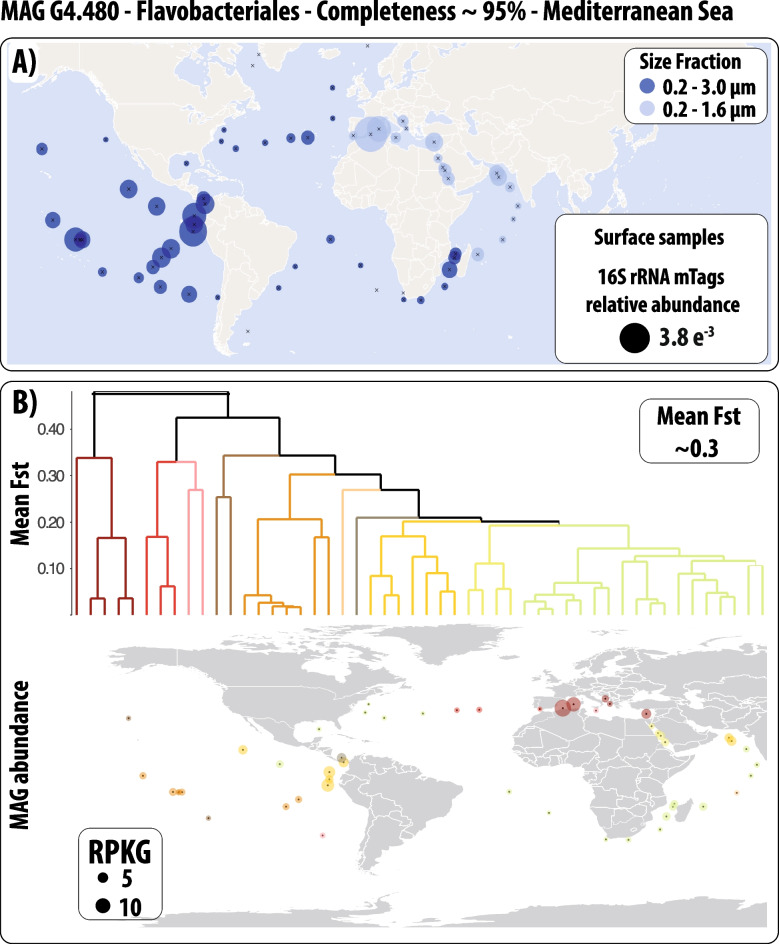
Accessing the population-level dimension of diversity in marine microbes using metagenomics. The figure aims to provide a simple example of the additional information on population structure that the metagenome-based population genomics approach can produce compared to 16S rRNA surveys. Here, I use the MAG G4.480 (uncultured Flavobacteriales, ~ 95% completeness, and < 10% contamination) that we retrieved from the Mediterranean Sea (LTER Blanes Bay Microbial Observatory; http://bbmo.icm.csic.es/). From this MAG, a fragment of the 16S rRNA gene (770 base pairs) was extracted and then used to estimate the MAG abundance in the global ocean and the Mediterranean Sea using the Ocean Barcode Atlas (OBA) [144] (https://oba.mio.osupytheas.fr/ocean-atlas/); results are shown in **A**. Only two 16S mTag [145] references from the OBA with > 99% sequence similarity with MAG G4.480 were considered (references AACY020490277.719.2228 and EF572435.1.1502; both Flavobacteriales, Flavobacteriaceae, NS5 marine group). Furthermore, only surface samples originating from two size fractions (0.2–1.6 and 0.2–3.0 μm) from the TARA Oceans cruise were included. In sum, in **A**, we observe the distribution of the MAG G4.480 as one single taxonomic entity. In **B**, the diversity within this entity is explored using metagenome-based population genomics (Fig. 3), and we notice that additional patterns emerge. In the upper section of **B**, the Fst values (measuring population differentiation) among the investigated stations were clustered, and different clusters, which may correspond to populations, were colored (Fst ~ 0.2 was used to delineate clusters). Note that some clusters correspond to geographic regions (**B**, lower section). For example, the clusters in the Mediterranean Sea, Red Sea, and Indian Ocean suggest that they could represent geographically delineated populations. These patterns are missed by the 16S rRNA gene (**A**). The abundance of the Mediterranean MAG G4.480 across the global ocean and the Mediterranean Sea based on metagenomic read recruitment is shown in the lower section of **B**. MAG abundances are indicated in RPKG (Reads Per Kilobase of MAG and Gigabase of metagenomic data). To obtain the Fst values and the abundances of the MAG G4.480 (**B**), we followed the procedure indicated in Fig. 3, which is partially implemented in POGENOM [104]. Only surface metagenomes from TARA Oceans with enough coverage (horizontal and vertical) of MAG G4.480 were used in downstream analyses, which explains the different numbers of stations included in **A** and **B**

Altogether, the previous studies reveal a considerable complexity in environmental microbial species at the population level, encompassing genomic diversity and structure, gene expression (including epigenetics), and fine-grained adaptations. We can now partially access this underexplored dimension of diversity through metagenome-based population genomics [103] and metatranscriptomics (Figs. 3 and 4). In addition, in multiple studies, selection seems to be central in structuring microdiversity, suggesting the fine-tuning of the ocean microbiome to environmental heterogeneity.

### Populations and contemporary evolution

Due to the large population sizes of many microbial species, the ocean microbiome could evolve faster than multicellular organisms with smaller populations [45]. However, there is still no precise estimate of the rate at which the ocean microbiome has evolved historically and how rapidly it may be evolving at present. This knowledge is essential in the context of global change, as evolutionary adaptation is one of the expected reactions of microbes to changing environmental conditions [40, 45, 46, 146]. Changing selective regimes are expected to select from the available genetic diversity of microbial populations and from emerging de novo mutations. Thus, the effectiveness of adaptation will partially depend on the genetic diversity and population size of each microbial species [45]. Dispersal rates will most likely influence adaptation to shifting environmental conditions and elucidating these rates for different species will be central to comprehending the ocean microbiome’s adaptation mechanisms. Widespread distributions across varying conditions [87], may confer more genetic flexibility to microbial populations. This could reduce the overall strength of purifying selection, leading to a richer reservoir of genetic variation that could enhance the adaptive capacity of populations. In addition, dormancy can weaken purifying selection [147], preserving genetic variants that may not be immediately beneficial but could become advantageous in different habitats.

The relative importance of the specific mechanisms underlying contemporary microbial evolution in the wild still needs to be elucidated. Thus far, microbial evolution experiments (in contemporary timescales) have indicated three major trends: (1) significant phenotypic innovations can emerge (e.g., new metabolisms, growth rates), (2) high levels of evolutionary *parallelism* (i.e., repeated evolutionary changes), and (3) emergence of population structure, such as genetically differentiated cell sub-groups [148, 149]. In contrast to laboratory experiments, relatively little is known about microbial evolution in natural habitats, and the interested reader is referred to Brennan and Logares for an in-depth discussion [45]. Here, I will briefly mention two examples from aquatic (non-marine) environments that illustrate the relevance of metagenome-based population genomics coupled with time-series metagenomics for understanding microbial evolution in the wild. These studies typically use a DNA archive, including samples from various time points, to track the evolutionary process. In the first example, Denef and Banfield investigated the evolution of a natural acidophilic biofilm over 9 years in Acid Mine Drainage (AMD) ecosystems [150]. An evolutionary rate of 1.3 × 10^−9^ substitutions per nucleotide per generation was estimated for one MAG, and further analyses showed how mutations could emerge and become fixed as a product of selection and drift. Given the extreme nature of AMD environments and the low immigration rates, it can be considered that mutations emerged in situ. Determining whether a mutation emerges in one location de novo or has arrived through immigration is a challenge in these types of studies.

Another study examined 30 bacterial MAGs that were derived from metagenomic samples collected over a 9-year period in a freshwater lake [151]. A large SNV heterogeneity was found between and among populations. This suggests varying mutation rates among species or populations or differences in immigration history. Newly arrived immigrants may exhibit more homogeneous populations as they have had less time to undergo diversification. SNVs frequencies showed marked changes over time in some populations. For example, in one population, most of the gene and SNV diversity disappeared during the investigated period, suggesting an ongoing genome-wide selective sweep [65], possibly the first observed in the wild [152]. In turn, another population displayed large, SNV-free genomic regions. These regions appear to have swept through the populations before the investigated period without removing diversity from other genomic areas, pointing to a gene-specific sweep [151].

The two previous studies exemplify the insights that can be obtained on contemporary microbial evolution in the wild through metagenome-based population genomics coupled with time series. As of now, this approach appears to remain underexplored in the context of oceanic studies. The connectivity of the ocean complicates the application of the approach, as it is difficult to disentangle mutations that originate in one location from those arriving via immigration. Nonetheless, temporal trends in SNV frequencies, as well as changes in both gene and SNV diversity, can offer valuable insights into the effects of shifting selective pressures induced by climate change on the ocean microbiome. This is of particular relevance in locations such as the Mediterranean Sea, which has experienced during the last years an increase in the frequency and intensity of marine heatwaves [153]. While these heatwaves have induced mass mortality events among multicellular marine organisms, their impact on the marine microbiome remains poorly understood.

### Microbial populations in a changing ocean

The ocean microbiome currently faces multiple challenges derived from anthropogenic-induced climate change. For example, sea-surface warming, decreasing O_2_ and increasing CO_2_ levels, acidification, changes in water circulation, changes in nutrient inputs, and other biotic factors (such as new parasites or predators) [146]. Thus far, relatively few studies have investigated the reaction of marine microbes to global change. Selective changes derived from global change can have significant consequences in microbial community structure, populations, evolution, and ultimately, in the biogeochemical cycles they mediate [146]. As a response to the changing oceanic conditions, microbes are anticipated to undergo shifts in their geographic distributions, alterations in community structure, modifications in gene expression—including epigenetic changes—, and adaptations to the new environmental conditions [41–43, 45, 46, 146, 154]. However, the relative significance of these mechanisms in shaping the overall response remains uncertain. Population genomics has the potential to provide new insights into the relative relevance of these processes in the reaction of microbes to a changing ocean.

Multiple studies have reported the effects of climate change on animal and plant communities [155], often highlighting a response lag known as climate debt [156–160]. This lag seems to be linked to the difficulties of animal and plant communities in keeping up with fast climatic changes [160]. It remains debated whether microbial communities are in equilibrium with current climate conditions or also experience climate debt [161]. Due to their short generation times, high dispersal capabilities [81], and the possible existence of widespread seed banks [87], microbes could display fast responses to climate change and track environmental variation. Yet, environmental legacy effects, that is, persistent influences of past environmental conditions on current communities, have been documented in soil and planktonic (rock pools) microbial communities in relation to drought, salinity, temperature, and rhizodeposits [162–165]. In particular, Ladau and colleagues reported a lag in the response of soil microbes from North America and the Tibetan Plateau to climate change, with current distributions of bacteria correlating with climatic conditions from ~ 50 years ago [166]. The observed delay likely results from the time needed for soil properties to adapt to climate change, limiting insights into the rapidity of prokaryotic reactions to these changes. The study predicts that the diversity of soil prokaryotes will increase significantly once it equilibrates with current climatic conditions, although forecasting the functional impacts of this change remains challenging [166]. Limited information exists on whether the global ocean microbiome's structure, encompassing populations and species, tracks current environmental variation or remains influenced by past conditions [167]. Understanding this is an important challenge for future studies aiming to comprehend better the links between ocean microbiome structure and ecosystem function, as well as the functional impact of changing community and population structure.

## Conclusions

Beginning in the 90 s with the onset of the “molecular revolution” and continuing into the 2000s with the advent of High-Throughput Sequencing technologies, omics approaches have significantly advanced our understanding of the ocean microbiome, revealing the various lineages it harbors, their distributions, and metabolisms. Specific markers, such as the rRNA gene, provided a clearer dimension of the diversity that is contained in the ocean microbiome. Yet, the rRNA gene normally underestimates or misses the dimension of diversity that is found within individual species (Fig. 4). So far, only a limited number of studies have delved into the population-level diversity of environmental microbes. Understanding the population diversity of microbes is fundamental for a better comprehension of ecosystem function and the adaptation of microbes to different niches. Isolating and culturing environmental strains has been one of the main obstacles in accessing the species-level diversity of microbes. Today, the use of metagenomics and metatranscriptomics allows us to investigate the diversity that is present within species, bypassing the need for culturing. Population-level studies have the potential to open a new chapter in environmental microbiology, deepening our understanding of the ocean microbiome's composition, configuration, ecological interactions, and intricate relationships with ecosystem functioning. This new knowledge will also be pivotal in the context of global change as we seek to comprehend the ocean microbiome’s resilience or vulnerability, as well as its potential impact on broader Earth system processes.

## Data Availability

Not applicable.

## Abbreviations

AMD: Acid Mine Drainage
FACS: Fluorescence-Activated Cell Sorting
Fst: Fixation Index
GSSS: Gene-Specific Selective Sweep
GWSS: Genome-Wide Selective Sweep
HGT: Horizontal Gene Transfer
HTS: High-Throughput Sequencing
MAG: Metagenome-Assembled Genome
Mb: Megabases
MVS: Metavariant Species
*N*: Census population size
*N*_*e*_: Effective population size
OTU: Operational Taxonomic Unit
RPKG: Reads Per Kilobase of genome and Gigabase of metagenomic data
SAAV: Single Amino-Acid Variant
SAG: Single-Amplified Genome
SNV: Single Nucleotide Variant
